# Pipe flow of pumping wet shotcrete based on lubrication layer

**DOI:** 10.1186/s40064-016-2633-3

**Published:** 2016-06-30

**Authors:** Lianjun Chen, Guoming Liu, Weimin Cheng, Gang Pan

**Affiliations:** State Key Laboratory of Mining Disaster Prevention and Control Co-founded by Shandong Province and Ministry of Science and Technology, Shandong University of Science and Technology, Qingdao, 266590 China; College of Mining and Safety Engineering, Shandong University of Science and Technology, Qingdao, 266590 China

**Keywords:** Wet shotcrete, Pipeline transport, Pressurel loss, Lubrication layer, Particles migration

## Abstract

Wet shotcrete can reduce dust and improve supporting strength, however, safe and efficient pipage is a key technical part of wet shotcrete process. The paper studied the pipe flow law of wet shotcrete based on lubrication layer by build the experimental pumping circuit of wet shotcrete that can carry out a number of full-scale pumping tests. The experimental results show there was a linear relationship between pressure loss and flow rate. Combined with the Buckingham rheological equation, the computing equations of the yield shear stress and plastic viscosity were deduced through linear regression. A simple analytical method allowing for a rough estimation of the pumping pressure was proposed and used when considering the lubrication layer of wet shotcrete in pipes. In addition, two kinds of particulate distributive models were established along the time axial to analyze the formation of lubrication layer which is related with particles migration. By computational fluid dynamics simulation, the lubrication layer thickness of different mix proportions was estimated. A new method for measuring the thickness of lubrication layer was proposed to verify it by binarization processing. Finally, according to the comparative analysis of experiments, simulation and computed value, it can be seen that the lubrication layer plays a key role in the process of wet shotcrete flow and with the increase of lubrication layer thickness pipe pressure declines gradually.

## Background

**Fig. 1:**
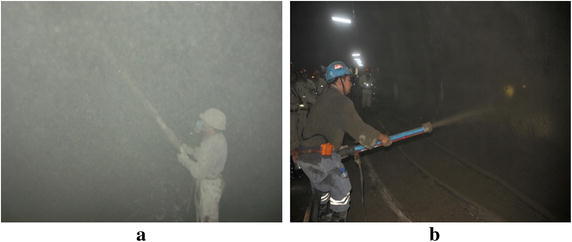
The Field situation comparison between dry shotcrete and wet shotcrete. **a** dry shotcrete, **b** wet shotcrete

A lot of mines are exploited with the mechanized underground operation beneath thick layers of rock-soil. The gangue haulages and undercut operations are supported under a combination of bolts and meshing covered in a layer of shotcrete to prevent the movement of surrounding rocks and protect the workers’ safety (Steward and Loggerenberg 2006). A great amount of dust produced in the process of traditional dry shotcrete has a strong impact on workers’ health, even causing the pneumoconiosis. The dry shotcrete of low strength may fail in supporting roadways under the action of rock burst. Luckily, the technology of wet shotcrete can solve the above problem. The field situation of wet shotcrete and dry shotcrete around spraying workers are shown in Fig. 1. And comparison of dust data is presented in Table 1.

**Table 1:** Dust concentration comparison between dry shotcrete and wet shotcrete

Measuring points	Wet shotcrete (mg/m^3^)		Dry shotcrete (mg/m^3^)	
	Total dust concentration	Respirable dust concentration	Total dust concentration	Respirable dust concentration
Around spraying workers	14.8	5.3	95.5	49

As shown in Fig. 1, total and respirable dust concentration of wet shotcrete are much lower than that of dry shotcrete obviously. Therefore, the application of wet shotcrete technology in mine support will be of great importance to decrease dust and improve supporting intensity. In China, shotcrete support of roadways is in the period from the “dry shotcrete” to “wet shotcrete”. However, the pipe blockage caused by the large resistance often occurs and affects the development of wet shotcrete. Hence, it is imperative to study the rheological behavior and on-way resistance in order to ensure the safety and effectiveness of wet shotcrete pipage.

The pipe deliver of wet shotcrete is commonly referred to as a full flow system in the concrete industry. The pipage system requires that the pump pressure should be equal or higher that the pressure loss generated by wet shotcrete flowing through the pipeline. The composition and workability of wet shotcrete are nearly similar to that of the self-compacting concrete (SCC). SCC requires relatively high pumpability, usually, its slump is larger than ordinary concrete, the continuous aggregate grading is strictly required, and the maximal particles diameter is less than one third of pipe diameter.

Many different hypotheses about the law of concrete flow behavior were proposed. Sakuta et al. (1989) and Lipovetsij (1963) regarded the nature of concrete friction stress as a constant along the pipeline for the given concrete and pipes:

1 $$ \tau_{f} = {\text{constant}} $$

However, it was generally accepted that wet shotcrete was a Bingham fluid which takes into account this yield stress and the plastic viscosity (Soualhi et al. 2015; Kaplan et al. 2005; Siedlarz and Gołaszewski 2015). The yield stress is the resistance to the primal flow, while the plastic viscosity is a measure for the resistance in Eq. (2).

2 $$ \tau = \tau_{0} + \eta_{p} \cdot \frac{dv}{dr} $$

where $\tau$ is the shear stress (Pa); $\tau_{0}$ is yield stress (Pa); $\eta_{p}$ is plastic viscosity (Bingham) (Pa s); $\frac{dv}{dr}$ is shear rate (s^−1^);

Due to structural breakdown and workability loss caused by chemical reactions, the rheological behavior of fresh concrete may deviate from the linear behavior. The rheological parameters of wet shotcrete are constants no longer (Wallevik 2003; Kirk et al. 2015; Roussel 2006). A different rheological model was widely applied, as is shown in Eq. (3).

3 $$ \tau = \tau_{0} + K \cdot \left( {\frac{dv}{dr}} \right)^{n} $$

where *K* is consistency factor (Pa s^n^); n is consistency index.

In order to describe the shear-thickening behavior, a modified Bingham model (Feys et al. 2008; Yahia and Khayat 2001) was proposed by the authors (Eq. 4). Ronny V applyed to the modified Bingham model and noted that shear-thickening had a great influence on the pressure loss.

4 $$ \tau = \tau_{0} + \eta \cdot \frac{dv}{dr} + c \cdot \left( {\frac{dv}{dr}} \right)^{2} $$

where $\eta$ is linear term (Pa s); c is second order parameter (Pa s^2^).

**Fig. 2:**
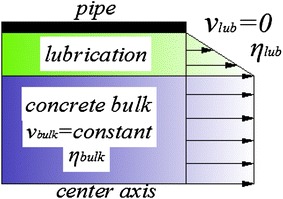
Schematic pattern of wet shotcrete flow in the pipe

These approaches have almost neglected the fact that a lubrication layer existed at the vicinity of the pipe wall. The lubrication layer was proposed by Aleekseev (1952) and Weber (1968). The shear of this layer allows the slipping of concrete and caused the pressure loss along the pipeline. The principle of slippage and lubrication layer is visualized in Fig. 2. In case of a lubrication layer, the velocity $v_{\text{lub}}$ at the wall is zero and the velocity gradient caused by the lower viscosity $\eta_{\text{lub}}$ in the lubrication layer is larger than that caused by the viscosity $\eta_{bulk}$ in the concrete bulk. When concrete is pumped, the yield stress of the concrete outside the lubrication layer is higher than the shear stress near the wall where the velocity $v_{bulk}$ of concrete bulk is a constant (Thrane 2007).

Jacobsen et al. (2009) conducted the experimental research with colored fresh concrete flowing after ordinary concrete to observe the flow conditions and demonstrated the lubrication existence at the interface between concrete bulk and the pipe. It was uncertain that the exact thickness of this lubrication layer in literatures was estimated to be between 1 and 5 mm (Austin et al. 2000; Crepas 1997). The redistribution of particles occurs within the pipe under the action of shear. The particles migration which moves from high shear rate regions to low shear rate regions was reported in literature (Choi et al. 2013), especially the migration of coarse particles may increase under the influence of the pipe wall.

The purpose of research and analysis was to study pipe flow law of wet shotcrete based on lubrication layer, mainly containing the pipe pressure calculation and the formation of lubrication. Considering that the detailed analysis of the formation and properties of this lubrication layer had not been carried out yet, this research of pumping wet shotcrete was conducted based on lubrication layer. Through Buckingham rheological equation, formulas of yield shear stress and plastic viscosity were deduced. The comparison analysis showed that the lubrication layer played a key role in the process of wet shotcrete flow. Moreover, its thickness mainly depended on mix proportion. Finally, the measures for easy forming lubrication were adviced.

## Materials and schemes

### Material

Three different kinds of common wet shotrete proportion were studied in this paper given in Table 2. The cement was ordinary portland cement with the density of 3120 kg/m^3^. The sand was a natural river sand with a density of 2560 kg/m^3^. The maximum coarse aggregate size was 10 mm less than pipe diameter. It was a limestone aggregate with density of 2670 kg/m^3^. Taking into account water absorption rate of sand and coarse aggregates, the amount of mixing water was corrected. A lignosulfonate-based water reducing admixtures (WRA) was used. Its dosage is shown in Table 2 marked as % WRA, meaning percentage of admixture relative to binder content (in weight). Each concrete was produced in a single horizontal-axis forced mixer on the scene. The mixing process was as follow: coarse aggregates and sand were mixed during 20 s; both of cement and fly ash were added during 20 s; WRA and water were added during the additional 2 min of mixing.

**Table 2:** Mix proportion

Materials	Design strength (C25–C35)		
Name of the series	Group 1	Group 2	Group 3
Cement (kg/m^3^)	401	430	410
Fly ash class F (kg/m^3^)	43	48	44
Water (kg/m^3^)	204	192	186
Sand (kg/m^3^)	876	889	887
Coase aggregate (kg/m^3^)	704	680	711
Lignosulfonate-based WRA (%)	0.3	0.25	0.25
Slump (mm)	110 ± 20	110 ± 20	100 ± 20

### Pumping system

**Fig. 3:**
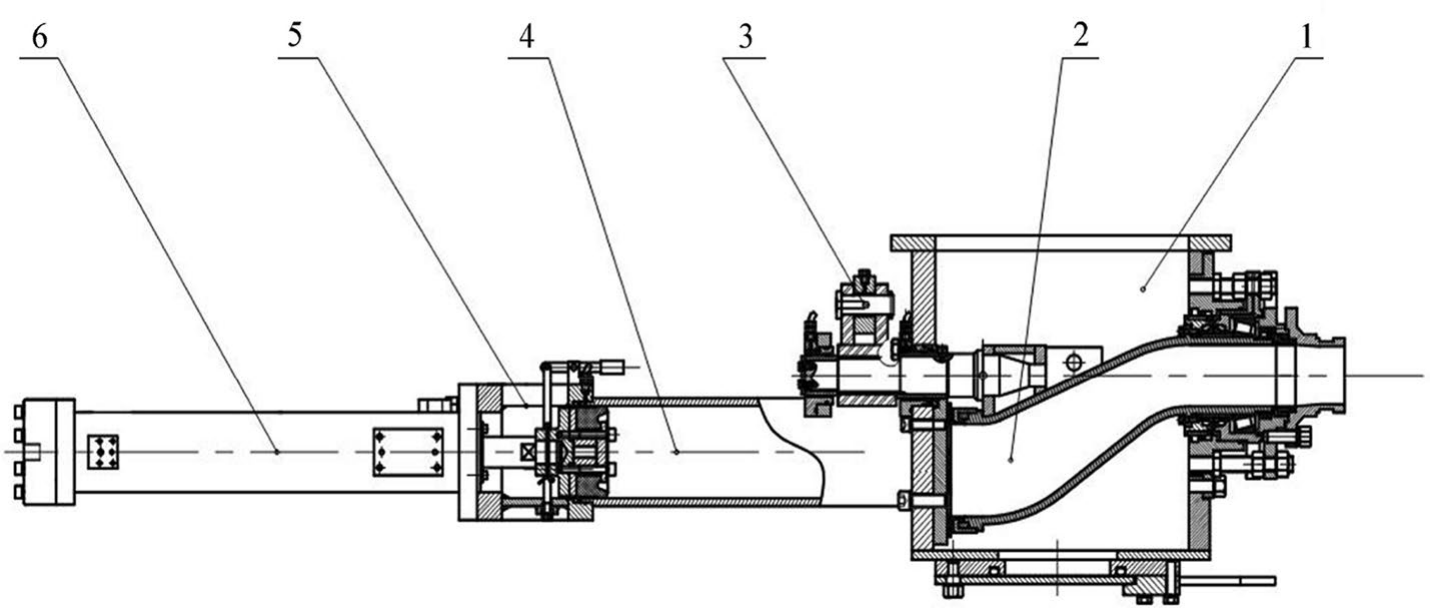
The pumping system of the new wet spraying integral machine. *1* hopper; *2* S valve group parts; *3* tilt cylinder; *4* working barrel; *5* water tank; *6* hydraulic cylinder

Experimental concrete transmission pumps (double piston pumps) were made of two paralleling pump cylinders as shown in Fig. 3. When the fresh concrete flowed into a pump cylinder, it was extruded from the other pump cylinder by strong hydraulic pressure. And fresh concrete were sent through S valve. The stroke rate of pump cylinders could be manually controlled by the handle of hydraulic pumps. And two hydraulic piston tilt cylinders were used to switch between the two pump cylinders in the S valve hydraulic system.

### Pipeline circuit

**Fig. 4:**
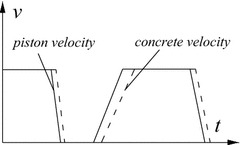
The characteristic of pumping velocity

The experiment was carried out with a horizontal pumping circuit of 100 m. Due to the workability of the piston pump, the phenomenon, fresh concrete being pushed alternately into the pipes and pulled from the pumping reservoir, may cause a sudden decrease and increase of pressure in one second. As shown in Fig. 4, it can be clearly seen in the measured pressure curve that the concrete velocity was always slower than piston velocity because the concrete was pushed by the alternate force from two pistons, concrete was passive while the pistons were active, and the fresh concrete may be compressed resulting in a part of loss of concrete velocity. The sound of alternate pistons could be clearly heard on site.

**Fig. 5:**
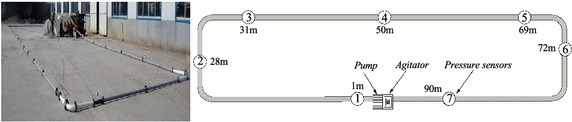
Overview of the experimental setup and the location of pressure gauges

The circuit was made up of steel pipes with an inner diameter of 64 mm and includes four 90° bends and 6 pressure sensors. The special concrete pressure sensor (Model EP3000) was used in tests and purchased from Xi’an Hehai Electronic Technology CO., LTD. The pressure sensor adopted the technology of the secondary encapsulation of silicone oil to prevent the coarse aggregates from breaking the membrance of the sensor. The detailed locations of the sensors are shown in Fig. 5. The first sensor was located at 1 m after the beginning of the circuit whereas the last one is located at 10 m before the end. The pressure sensors were connected to a data-acquisition system which registered the local pressure. With these sensors, the pressure at difference locations can be measured and the pressure loss per unit of length can be calculated.

## Experimental results and analysis

### Flowing behavior of fresh wet shotcrete

**Fig. 6:**
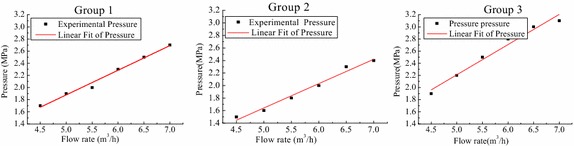
Pressure-flow rate relationship in the 100 m full-scale test

The pumping tests of each mix proportion were performed at five discharge rates available on the pump. By taking the pressure given from the No. 1 sensor as the pumping pressure, one obtained an experimental point for each flow value. As shown in Fig. 6, there was a nearly linear relationship between pressure and the flow rate by using linear interpolation fitting. It can be seen that the lower the flow rate is, the smaller the pipe pressure is. In the pumping process, the flow rate could be smaller for reducing the pressure loss under the condition of guaranteeing the pumping success.

Different groups of mix proportion had different pressure at the same position whose value was highest in Group 3 and lowest in Group 2. The pressure value mainly depends on the rheological parameters (*i.e.* mix proportion) in the case of the same flow rate. To analyse the reason, literatures (Choi et al. 2013; Ingber et al. 2009; Lu et al. 2008) indicated that a redistribution of particles occurs in the pipe under the action of shear, particles migrating from high shear rate regions to low shear rate regions, which is relative to the formation of lubrication (next “The formation of lubrication layer” section would focus on the lubrication formation). Literatures (Spangenberg et al. 2012; Myoungsung et al. 2013) also reported that the origin of shear induced by particle migration is generated by the competition between gradients in particle collision frequency and gradients in viscosity of the concrete within the pipe. As discussed above, the shear is affected by the particles and material viscosity.

The shear and viscosity are directly influenced by mix proportion (Xiao and Yue 2011). It was shown in Duan (2011), Liu (2005) that with the increasement of water-binder ratio, the yield shear stress presents the declining trend while the plastic viscosity is irregularity, and with the sand rate increasing, the trend of yield shear stress firstly go up and then down. It is also obvious that both of shear and viscosity contribute to the pipe pressure (Wallevik 2003; Kirk et al. 2015; Feys et al. 2008; Yahia and Khayat 2001).

Therefore, the pressure was influenced indirectly by mix proportion. The rheological parameters of three fresh concrete were measured by fresh concrete rheometer (Model eBT2, purchased from Germay Schleibinger company), the results are shown in Table 3.

**Table 3:** Rheology parameters

Type	Group 1	Group 2	Group 3
Plastic viscosity (Pa s)	15	12	26
Yield stress (Pa)	85	113	127

**Fig. 7:**
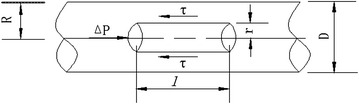
Fresh wet shotcrete flow in the pipeline

It is known that in the process of pumping, the wet shotcrete, a kind of composite material which includes water, cement and aggregates as the main components, moves in the pipe as a large bulk. In a point of suspension, solid particles of shotcrete suspense concentratedly in a viscous liquid (*i.e.* paste or mortar). Under the action of pump push force, the unit equilibrium analysis of wet shotcrete in a pipe is shown in Fig. 7.

5 $$ \pi r^{2} \cdot \Delta p = 2\pi r \cdot \tau \cdot l $$

6 $$ \tau = r \cdot \frac{\Delta p}{2l} $$

Based on the fact that fresh concrete was analogous to a general Bingham body, the pumping of wet shotcrete was regarded as a sort of flowing of Bingham liquid along pipelines. In accordance with the derivation method of Eq. (2), Buckingham rheological equation was derived as follows:

7 $$ \tau = \frac{4}{3}\tau_{0} + \eta \cdot \left( {\frac{8v}{D}} \right) $$

where η is the viscous constant (Pa s) and *v* is the slip speed (m/s), D is the pipe diameter.

As the profiles of pressure are shown in Fig. 6, there was the linear relationship between the axial pressure variation $\Delta p$ and flow rate for an element in the pipe whose length was *l*. In order to describe the mathematical relationship between the stress and the speed, another linear regression Eq. (8) was done based on Eq. (7)

8 $$ \tau = a + b \cdot v $$

The linear regression maked it possible to find the rheological parameters from Eq. (7)

9 $$ \left\{ \begin{aligned} \tau_{0} = \frac{3a}{4} \hfill \\ \eta = \frac{rb}{8} \hfill \\ \end{aligned} \right. $$

### The formation of lubrication layer

Indeed, the loss of pressure is located on the lubrication layer which consists of cement paste or mortar, as the coarse aggregates move away from the zone with shear-induced particle migration. As is shown in Fig. 2, the entire velocity variation between pipe wall and concrete bulk is concentrated in this layer. In order to explore the formation mechanism of the lubrication layer, two kinds of particulate distributive models are established along the time axial: slurry cementing model and capillary water coupling model.

1. Slurry cementing model. It is known that the concrete particles evenly distribute in the hopper before pumping, and the particles are uniformly filled in free cement mortar whose connection among particles is slurry cementing model. At the same time, the force between liquid and particles is greater than or equal to the force between particles $F_{lk} \ge F_{kk}$, as is shown in Fig. 8a

**Fig. 8:**
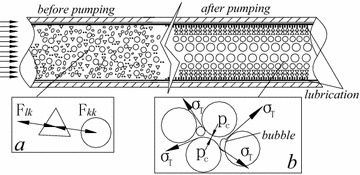
Physical model of particle distributein pipe. **a** slurry cementing model, **b** capillary water coupling model
2. Capillary water coupling model. In the process of pumping, the uniform distribution of particles is broken by the tip flow of cement paste and by inertia of particles motion at the pipe wall, relative with the viscous drag of the inserted cement paste, forcing the coarse particles to move towards the pipe center. In the proper distribution proportion, the kinetic particles suspension is caused by the sum of lift force and buoyancy which is greater than gravity in the pipe. Therefore, the free slurry is pushed towards the bottom half of pipes to form lubrication. However, due to the operating characteristic of the piston pump, the flowing speed of wet shotcrete in the pipe is slower than that of piston pump and the particles speed in wet concrete is not always at the peak of pumping speed (Fig. 4). The particles whose gravity is greater than buoyancy begin to sink in the pipe when they lose lifting force because of low or no speed leading to the formation of the lubrication layer in the upper half of pipes. At the same moment, the particles which have concentrated in the center of pipes keep on suspending at the action of capillary water tension. Therefore, the stability of pumping is beneficial for the formation of lubrication, while the longer machine halt or bigger pumping pressure is prone to breaking the normal particles migration for forming lubrication.

In other words, the formation of lubrication layer is caused by the particles migration. The force between liquid and particles is transformed to the capillary water tension. The skeleton of wet shotcrete is filled with the liquid and air, where there is a surface tension at the interface of liquid and air bubble, as is shown in Fig. 8b. At the same time, the liquid in the skeleton void changes to be the capillary edges liquid under the pull $\sigma_{T}$, and the particles is forced to aggregate under the action of the pressure $P_{c}$.

### Pipe pressure calculation based on lubrication layer

The rheologic material of the lubrication layer is the one of the constitutive mortar. There is a simple approach that can capture the concentration of shear in the fluid lubrication layer (Myoungsung et al. 2013). The shear rate in the lubrication layer can be estimated as

10 $$ \frac{{dv_{lu} }}{dr} = \frac{{V_{0} }}{{\delta + R\eta_{lu} /2\eta_{bulk} }} $$

where $v_{lu}$ is the average velocity of the lubrication layer, δ is the lubrication layer thickness, $\eta_{lu}$ is the plastic viscosity of the mortar in the lubrication layer, $\eta_{bulk}$ is the plastic viscosity of concrete in the bulk, *V*_*0*_ is the average concrete velocity.

In this shear rate range, the contribution of the yield stress of the mortar can be neglected and the average shear rate in the lubrication layer can be approximated as $\frac{{dv_{lu} }}{dr} = \frac{\Delta p \cdot R}{{2L\eta_{lu} }}$, so the pressure gradient can be computed as:

11 $$ \frac{\Delta p}{L} = \frac{{2\eta_{lu} V_{0} }}{{R(\delta + R\eta_{lu} /2\eta_{bulk} )}} $$

where $\eta_{lu}$ can be calculated from the Eq. (9). The size of particles should be considered when estimating $\eta_{bulk}$ (Mahmoodzadeh and Chidiac 2013), so the approach has been adopted in this formula,

12 $$ \eta_{bulk} = \eta_{i} \cdot \left[ {y^{3} \frac{{4(1 - y^{7} )}}{{4(1 + y^{10} ) - 25y^{3} (1 + y^{4} ) + 42y^{5} }}} \right] $$

where $\eta_{i}$ is instrinsic viscosity, y is a function of concentration $\varphi$, $\varphi_{m}$ is the maximum packing density.

13 $$ y(\varphi ) = (\varphi /\varphi_{m} )^{1/3}$$

## Numerical simulation and verification

The lubrication layer facilitates the flowing of fresh wet shotcrete through pipes. In order to predict the thickness of lubrication layer, the computational fluid dynamics (CFD) simulation and comparative analysis with experiment data were therefore used to solve this complex problem. In the stabilization stage of pumping, the concrete flow was nearly continuous and the interaction between particles were slight. Therefore, in simulation, the fresh concrete was simplified and regarded as single phase fluid. Therefore, the computational modeling techniques could simulate concrete flow by using a single phase fluid method with the commercial CFD code Fluent (Gram and Silfwerbrand 2011; Fluent Inc 2011). The parameters of fresh concrete for simulation are shown in Table 4.

**Table 4:** Parameters of fresh concrete for simulation

Material no.	Phase	Density (kg/m^3^)	Viscosity coefficient (Pa s)	Thickness preseted of lubrication layer (mm)
Group 1	Single phase	2220	15	1–5
Group 2	Single phase	2239	12	1–5
Group 3	Single phase	2238	26	1–5

**Fig. 9:**
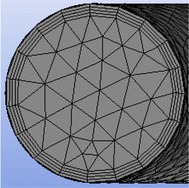
Cross section including the lubrication layer

Those following boundary conditions were used as follows: the inlet velocity of wet shotcrete was set according to the flow rate and the outlet pressure was fixed at the atmospheric pressure. No slip conditions were applied for the pipe interface. The meshing zone was divided into a lubrication layer and a bulk zone (Fig. 9).

**Fig. 10:**
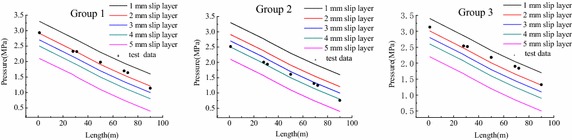
Pressure variation for different lubrication layer thicknesses

We considered the case of a 100 m pumping circuit with a 7 m^3^/h flow rate and the varied lubrication layer from 1 to 5 mm. Then we compared the simulation pressure profile with the experimental pressure along the pipeline (Fig. 10).

**Fig. 11:**
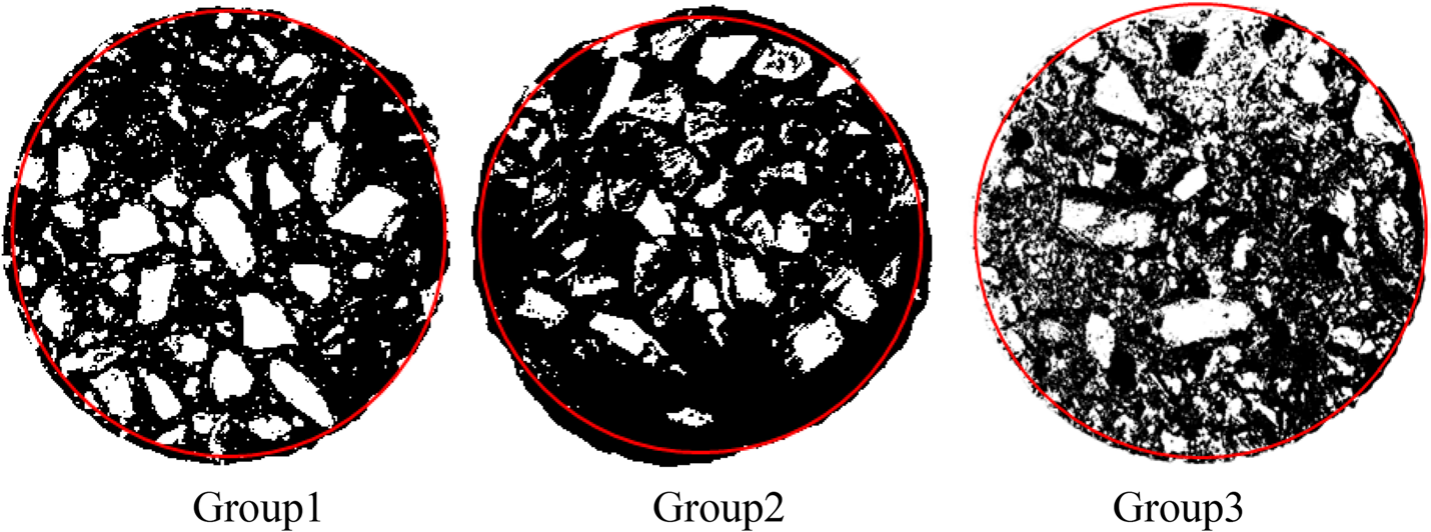
Binarization processing of the cross-sections of three concretes

The numerical simulation results of different lubrication layers and the pressure distribution along the pumping circuit for the three tested concretes are shown in Fig. 10. The different thickness of lubrication layers had different numerical simulation results that the thicker the lubrication layer is, the smaller the pipe pressure is. The simulated pumping pressure of 1 mm lubrication layer was approximately 1.7 times higher than those of 5 mm lubrication layer. It could be seen that no or less lubrication layer caused by unreasonable mix design may result in high pipe pressure. If the pumping pressure required by pumping wet shotcrete exceeds the capacity of the high pressure pump, pipe blockage will happen. The best agreements of Group1, Group 2 and Group 3 were obtained respectively for the layer thickness of 2, 3.5 and 1.5 mm. To verify the thickness simulated, a new method for measuring the thickness of lubrication layer as follow: cutting three kind of flowing concrete in pipe along cross-section after hardening; photographing the cross-section and binarization processing as shown in Fig. 11; measuring the distance of ten aggregates that are most close to the outer boundary from the outer boundary of cross-section; finally, calculating the average value of the distances as the the thickness of lubrication layer as shown in Table 5. It can be seen that the tested value is similar to the simulated value.

**Table 5:** Calculation of measured thickness and omparation with simulation

No.	1	2	3	4	5	6	7	8	9	10	Average value of test	Simulated value
Group 1 (mm)	1.8	0.2	2.4	3.3	0.7	2.6	2.1	1.4	3.1	1.7	1.93	2
Group 2 (mm)	4.6	3.3	0.4	4.7	5.3	2.3	3.3	3.6	4.6	2.5	3.46	3.5
Group 3 (mm)	0.2	1.8	0.9	1.5	0.6	2.5	1.8	2	1.7	1.3	1.43	1.5

**Fig. 12:**
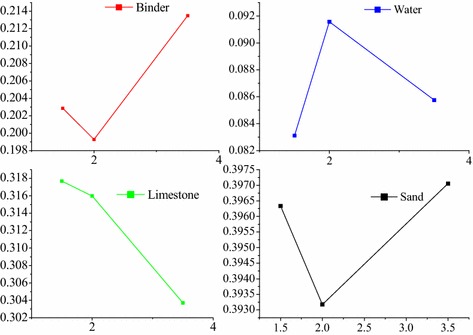
Relationship between thickness of lubrication layer and content of each material, X indicates the thickness of lubrication layer (mm), Y indicates the content of each material relative to concrete (%)

In order to deeply study, the relationship between thickness of lubrication layer and content of each material was analysed as shown in Fig. 12. There were not strong dependency between thickness of lubrication layer and content of binder, water and sand, excepting limestone that showed the declining trend with increasing lubrication thickness. However, it can be seen that the content of binder and sand was largest in concrete than that of others, which may explain that the more cement and sand, less coarse aggregate, are beneficial for the formation of lubrication layer. We also gained that the thickness of lubrication layer increases with the plastic viscosity increasing from Table 3. In addition, the thickness of lubrication layer may be effected by the aggregate gradation, the proposed point should be future studied next step.

**Fig. 13:**
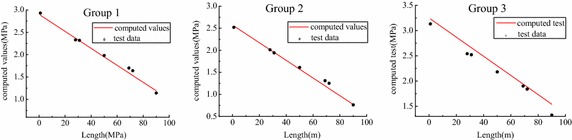
Comparation between computed and tested value

Combined with the thickness of lubrication layer, 2, 3.5 and 1.5 mm, from the numerical simulation and measured value, the pressure distribution calculated from the pressure gradient formula (Eq. 11) was plotted in Fig. 13. There was a better agreement between the computed and tested value, and the discrepancies between them could be seen: the computed value was similar to the experimental data in Group 2, while there were the most discrepancies in Group 3 that the calculated results were entirely greater than the experimental data. According to the analysis, the discrepancies of three comparative groups may be caused by the different mix proportion.

## Conclusions

In order to achieve high quality wet shotcrete for the tunnel mine support and understand the pumping process of wet shotcrete, the pumping experiments that can carry out a number of full-scale pumping tests were studied in this paper. The experimental results showed that there was the linear relationship between pressure loss and flow rate. The computing equations of the yield shear stress and plastic viscosity were deduced by linear regression with Buckingham rheological equation. In addition, in order to analyze the formation of lubrication layer relating with particles migration, two kind of particulate distributive models were established along the time axial: slurry cementing model before pumping and capillary water coupling model in the process of pumping. By the computational fluid dynamics CFD simulation, the thickness of the lubrication layer was respectively found to be around 2, 3.5 and 1.5 mm in Group1, Group 2 and Group 3. A new method for measuring the thickness of lubrication layer was proposed to verify it by cutting the harden concrete and binarization processing. Two of test and simulation showed similar results of thickness. This layer thickness mainly depended on the concrete mix design in Group 2 was extremely suitable for the wet shotcrete flow in pipes.

On a basis of the simulation results, the computed method of lubrication layer were obtained from the rheological characteristics of wet shotcrete. The pumping pressure was estimated with various thickness of lubrication layer, which obtains a good result. According to the comparative analysis, the lubrication layer played a key role in the process of wet shotcrete flow and the pipe pressure gradually declined as the lubrication layer become thick. In conclusion, for improving the efficiency of lubrication formation and pumpability, the wet shotcrete should be pumped stably at relatively low flow rate, and the mix proportion should be adjusted with relatively increasing cement and sand content, reducing the content of coarse aggregate in a reasonable range.

## Abbreviations

$\tau$: the shear stress (Pa)
$\tau_{0}$: yield stress (Pa)
$\eta_{p}$: plastic viscosity (Bingham) (Pa s)
$$ \frac{dv}{dr} $$: shear rate (s^−1^)
*K*: consistency factor (Pa s^n^)
n: consistency index
$\eta$: linear term (Pa s)
c: second order parameter (Pa s^2^)
$v_{\text{lub}}$: the velocity at the wall
$\eta_{\text{lub}}$: viscosity in the lubrication layer
$\eta_{bulk}$: the viscosity in the concrete bulk
$v_{bulk}$: the velocity of concrete bulk
*v*: the slip speed (m/s)
D: the pipe diameter
δ: the lubrication layer thickness
*V*_*0*_: the average concrete velocity
$$ \eta_{i} $$: instrinsic viscosity
y: a function of concentration $\varphi$
$\varphi$: the concentration of concrete
$\varphi_{m}$: the maximum packing density
